# Impact of the COVID-19 pandemic on the regular follow-up and outcomes of patients with chronic myeloid leukemia in chronic-phase

**DOI:** 10.3389/fonc.2022.994101

**Published:** 2022-10-06

**Authors:** Umut Yılmaz, Selin Küçükyurt, Sertaç Tunç, Ahmet Emre Eşkazan

**Affiliations:** ^1^ Division of Hematology, Department of Internal Medicine, Cerrahpaşa Faculty of Medicine, Istanbul University-Cerrahpaşa, Istanbul, Turkey; ^2^ Cerrahpaşa Faculty of Medicine, Istanbul University-Cerrahpaşa, Istanbul, Turkey

**Keywords:** chronic myeloid leukemia, CML, COVID-19, molecular monitoring, response

## Abstract

**Introduction:**

COVID-19 immediately became a major consideration in the management of chronic myeloid leukemia (CML). The influence of such considerations on viral transmission rates and leukemic control remain to be explored. We conducted this study to identify these alterations and to investigate their clinical consequences.

**Methods:**

This was a cross-sectional study, performed at a single institution on CML patients who were interviewed with a survey. We compared variables concerning new attitudes in the pandemic era between the 12-month periods before and after the pandemic onset. Outcome data were attained from the hospital archives.

**Findings:**

The number of patients receiving regular outpatient care for CML in chronic phase was 210, 91% had achieved at least major molecular responses. We assessed survival, progression, number of clinical visits of all, performed the survey on 89% and evaluated molecular responses on 86.6% of these patients. The frequency of clinical and molecular monitoring was significantly reduced during the pandemic deviating significantly from the guidelines. Frequency of death, progression, loss of molecular response was not significantly increased during the pandemic era despite a few cases where the delay in assessment possibly played a role in the unfavorable outcomes. There were no COVID related deaths or disabilities.

**Conclusion:**

The case-based untoward events would have probably been better managed with a more efficient communication web between patients, hematologists, and the laboratory. Therefore, it seems reasonable to consider whether such communicative paths are functional before giving up on the set schedule of CML management at times of uncertainty.

## Introduction

Chronic myeloid leukemia patients in chronic phase (CML-CP) treated with tyrosine kinase inhibitors (TKIs) are expected to be protected from disease progression while maintaining a reasonably undisturbed quality of life. However, the achievement and maintenance of this stable state are not straightforward. It requires specialized care through scheduled clinical visits for disease and adverse event (AE) monitoring to modify treatment. The health status of a CML patient is also subject to changes due to emerging co-morbidities with age, which may necessitate the treatment adjustments. The European LeukemiaNet (ELN) recommends clinical visits every three months for CML-CP patients enjoying a stable course and more frequent evaluation to others (1).

The COVID-19 pandemic, along with its overall detrimental impact on human activity, has interfered with the CML management agenda. Widespread recommendations to delay elective clinical interventions, travel limitations, quarantine applications, limitation of health care capacity, and personal preferences of patients to avoid crowded environments have contributed to reduced hospital visits. Whether this disturbed schedule in CML management is detrimental or not is unknown; however, disease progression, TKI incompliance, and TKI toxicity are concerning in such unsupervised patient population.

## Materials and methods

In this study, we screened all the CML patients treated in our institution taking two reference time points, March 2019, and March 2020. We gathered data regarding the medical history, CML course, and molecular responses before the pandemic from the patient files in our archives. We performed a face-to-face survey for patients who presented to the clinic during the study period to gather data on the effects the pandemic had on TKI adherence, whether they went through COVID-19, noted their molecular responses, screened for possible TKI-related symptoms. Patients who did not present to the clinic were contacted by telephone for attaining similar information and endorsing patients to apply to the clinic for regular clinical assessment and molecular testing. We used the independent samples t-test, chi-square test, and one way ANOVA for analysis, considering *p*<0.05 statistically significant.

## Results

**Figure 1:**
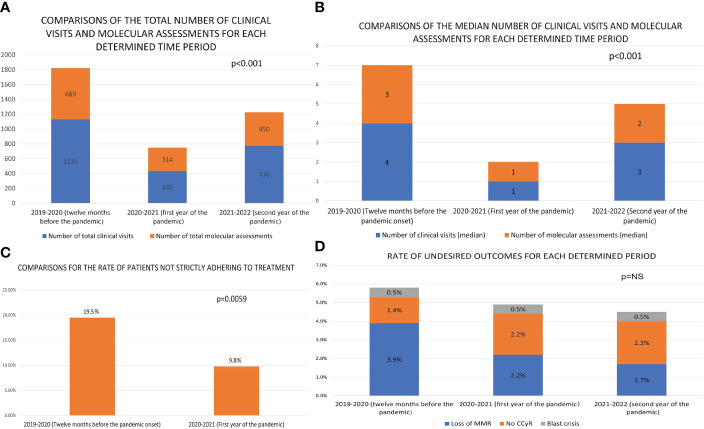
The comparisons of selected features between the twelve months prior to the pandemic and the first twelve months of the pandemic [**(A)** The comparison of the total number of clinical visits and molecular assessments of CML-CP patients at the institution. **(B)** The comparison of the median number of clinical visits and *BCR::ABL1*IS PCR testing for follow up of CML-CP patients at the institution. **(C)** The comparison of the ratio of TKI-incompliance among CML-CP patients at the institution. **(D)** The comparison of the number and ratio of patients who have lost major molecular response (MMR) and those who have lost or not achieved the equivalent of complete cytogenetic response (NS, not significant)].

There were 210 CML-CP patients receiving regular medical care from the study center at the time the pandemic began in Turkey (March 2020), when 90.5% had achieved at least major molecular response (MMR), continuing treatment in a stable state. The median time since CML diagnosis was 10.1 years. Most patients (81.9%) were under imatinib therapy, and only 4% of patients had not yet reached complete cytogenetic response (CCyR) (**Table 1**). The total and median numbers of clinical visits and *BCR::ABL1* on international scale (IS) QRT-PCR assessments per patient was significantly reduced in the first twelve months of the pandemic compared to the twelve months before its onset (1131/4 vs. 435/1 and 689/3 vs. 314/1, *p*<0.001 for both, **Figures 1A, B**). These numbers improved in the second year of the pandemic to 776 total number of clinical visits and 451 molecular assessments; however, they remained significantly lower compared to the pre-pandemic level. Twelve months into the pandemic, 206 patients from the initial cohort (98.1%) were alive, and 205 (97.6%) were continuing TKI therapy. One patient died due to blast crisis, and three succumbed to non-CML-related causes. Three more patients died in the second year of the pandemic, one due to blast crisis and the other two were non-CML related. The median number of weeks for the delay of the first clinical visit after the pandemic onset was 32, and 19% of patients had no single clinical assessment in the initial twelve months of the pandemic.

**Table 1:** The demographic and baseline features of the cohort at the onset of the pandemic.

Parameter	(n=210)
**Median age, years (range)**	56 (26-92)
**Sex, n (%)**	
Male	114 (54)
Female	96 (46)
**Sokal risk scores, n (%)**	
Low	125 (58)
Intermediate	63 (29)
High	8 (4)
NA	20 (9)
**ELTS risk scores, n (%)**	
Low	138 (66)
Intermediate	43 (20)
High	12 (6)
NA	17 (8)
**Median duration of follow-up, months (range)**	121 (12-276)
**Currently used TKIs, n (%)**	
Imatinib	171 (81.5)
Dasatinib	19 (9)
Nilotinib	16 (8)
Bosutinib	1 (0.5)
Ponatinib	2 (1)

(NA, not available).

We performed the survey on 187 (90%) patients at the end of the first year of the pandemic. The frequency of patients claiming strict adherence to TKI therapy was higher when asked for the period after the pandemic onset compared to the year before (88.8% to 78.1% *p*=0.006 - **Figure 1C**). None of the interviewed patients reported concerns that TKIs would place them at a higher risk for severe COVID-19, and no patient faced difficulty in accessing TKIs during the pandemic. Twenty-one patients experienced COVID-19 in the first twelve months of the pandemic, with only four of these interrupting TKI therapy during the infection (for concerns of drug-drug interactions). None of the 21 cases died due to COVID-19 or required ventilatory support. COVID-19 cases from the second year of the pandemic were not available due to changes in public health reporting systems and newly placed limitations on data access. However, none of the three patients from the initial cohort that died in the second year of the pandemic were going through COVID-19 at the time of their demise, confirming that there were no COVID-19 related deaths in the second year of the pandemic.

The *BCR::ABL1*IS QRT-PCR results within three months of the pandemic onset and 12-15 months into the pandemic were available in 182 patients. Four of these 182 had lost MMR, one had lost CCyR, and two had progressed to blastic phase (**Figure 1D**). The number of these events was similar when compared to the data of the same cohort (plus three patients that had progressed or passed away before March 2020) from the twelve months (*p* = 0.26) before the pandemic onset, and the second year of the pandemic (2 loss of MMR, 1 blast crisis, 1 lost CCyR, *p* = 0.78). The genetics laboratory hands the PCR results only to patients and the results are not digitally available for physicians to follow. The patients with blast crisis and loss of CCyR in the first year of the pandemic were all in warning status (ELN2020) at their last assessment; however, they had not attended the clinic to discuss these results with their physicians. While performing the survey, we discovered that three patients had undergone dose reductions or termination of their TKI therapy due to presumed AEs (dyspnea, rash) without the supervision of a hematologist. We identified no case of an attempt at TFR in the first year of the pandemic.

Two patients who experienced blast crisis were under imatinib therapy at the time of transformation. Both patients were highly incompliant throughout their disease course with irregular TKI use and less than yearly clinical visits for disease monitoring. Both had previously documented episodes of molecular relapse due to incompliance, followed by improvements by implementing drug adherence. One of these was a 37-year-old male with CML-CP for 17 years. He presented with blast crisis in the third month of the pandemic. Y253F ABL mutation was detected. He achieved complete remission with two cycles of decitabine (10mg/m^2^ for 10 days) and dasatinib (100mg/day) combination and received hematopoietic stem cell transplantation from an HLA-matched sibling following his return to a second chronic phase. However, he died of bacterial sepsis in the first month of the transplantation. The other patient was an 80-year-old male with CML-CP for 8 years. He was intolerant to second generation TKIs, mainly due to cardiac comorbidities and chronic kidney disease and was resuming imatinib despite failing to achieve CCyR. The patient presented with blast crises in the 15^th^ month of the pandemic. M244V ABL mutation was detected. Bacterial and fungal infections interrupted salvage therapy with decitabine and dasatinib resulting in dose reductions and delays. Initially a short-lived partial response was achieved; however, he died with progressive leukemia within six months of blast crisis.

## Discussion

The study documents the disrupted schedule for clinical assessment and molecular monitoring of CML-CP patients in the first year of the COVID-19 pandemic. Findings are suspicious of a negative impact of this disrupted schedule on three cases whose warning status was not presented to the clinic in time and the three patients whose TKI therapy was inappropriately altered. The findings are not alarming for an exceptionally high risk of severe COVID-19 infection in CML-CP patients under TKI therapy.

An online survey from the United Kingdom investigating the changes in the mode of care for CML patients, identified 8.3% of the responders had faced difficulty obtaining TKIs during the pandemic, mainly due to drug unavailability (2). The national policy of the Turkish Republic during the initial period of the pandemic allowed patients with chronic diseases to receive their previously prescribed regular medication without the need for new prescriptions. We consider this policy was crucial in ensuring TKI adherence, especially during the early days of the pandemic when the uncertainty of how to prevent contracting COVID-19 drove most of the population into a sense of panic, with some taking extreme precautions such as avoiding all human contact and never leaving their house. The elective clinical procedures and appointments were also mostly delayed in the first year of the pandemic due to the uncertainty of transmission rates, at-risk populations, and the unavailability of the vaccine.

Whether CML-CP patients were under a higher risk for severe COVID-19 due to the disease itself or its treatment were addressed in a few studies earlier during the pandemic (3–7). The advanced age of most CML-CP patients, pulmonary AEs of dasatinib, drug-drug interactions between TKIs and COVID-19 treatments, thrombogenicity of ponatinib and nilotinib were especially concerning at the onset of the pandemic. Our group was among the earliest contributors of COVID-19 experience in CML-CP with a five-patient series, who recovered without serious respiratory compromise (5). This study provides an expansion of the initial observations on five patients to 21 patients, with similarly benign course in all cases. The Italian group reported 12 deaths among 217 CML-CP patients (5.5%) who had undergone COVID-19. Also addressing similar issues with this study, reporting that 24% of CML-centers altered their molecular monitoring schedule with more than half suspending the strategy for treatment free remission, citing difficulties in frequent monitoring (4). Other studies presented a slightly more pessimistic outlook with 11-13% mortality rates for COVID-19 in CML-CP patients in the first year of the pandemic (6, 7). Differences of age, screening practices, applied preventive methods for avoiding COVID-19 transmission, ethnicity, and comorbidities may be among the variables responsible from the discordance of mortality rates between studies. The COVID-19 vaccines were also studied in CML-CP and demonstrated robust immunologic activity in both humoral (8) and cellular (9) components following vaccination, dismissing concerns for impaired vaccine efficacy. Overall, the reviews concerning the issues mentioned above leave the impression that the presence of CML-CP was probably not a significant independent risk factor for severe COVID-19 or vaccine failure (10, 11). Although the study is not powered to address issues of COVID-19 mortality, our finding that none of the seven deaths in two years was attributed to COVID-19 may reopen the debate on the view that TKIs have a potential antiviral activity as postulated in earlier studies (12).

Attempts at treatment free remission (TFR), which is widely regarded as a new goal in CML management were hardly feasible during the initial stages of the pandemic as it necessitates at least monthly follow-up in the initial months (12, 13). The flexibility of the monitoring schedule for TFR is not a studied topic, impelling us to consider that such an ambitious goal can be postponed during states of emergency.

The study has two major limitations. First, the follow-up period is currently too short to reach a conclusion. Even if the disrupted clinical assessment schedule, drug non-adherence, and improper management of AEs during the pandemic are to have an impact on the control of CML-CP or patient outcomes, these would likely be only apparent with longer follow-ups due to the chronic nature of the disease. The second is the missing data. Although we were able to assess 90.0% of the cohort for well-being, hematological remission, drug adherence, and 86.7% with pre- and post-pandemic molecular analysis; the remaining patients may represent an especially incompliant subpopulation as their tenacious attitude towards delaying clinical and molecular assessment is suggestive of. Non-inclusion of such an incompliant population constitutes a potential bias, a warning to interpret this study’s findings with caution. As most of the issues mentioned above have resolved now for the COVID-19 pandemic, we consider the events that unfolded during this pandemic, especially that of the initial stage of uncertainty, need to be documented, long-term impacts sought and discussed as if they were a rehearsal of similar disruptions of daily life we may face in prospect.

## Conclusion

The lessons learned from the experience with COVID-19 may be valuable during a possible future pandemic, war, natural disaster, or economic depression when the initial uncertainty and panic will likely be similar. The case-based untoward events uncovered through this research would have probably been better managed with a more efficient communication web between the patients and the hematology unit. This could be achieved through e-mail groups, phone lines, social media, etc., depending on the center’s preference. On the other hand, a more collaborative clinical and laboratory communication would have also been highly beneficial, especially to discuss *BCR::ABL1* levels without the need for patients to attain the results and present them to their physicians. Therefore, it seems reasonable to consider whether such communicative paths are set and functional before giving up on the set schedule of clinical and molecular follow-ups at times of uncertainty and disrupted order.

## Data availability statement

The original contributions presented in the study are included in the article/supplementary material. Further inquiries can be directed to the corresponding author.

## Ethics statement

The studies involving human participants were reviewed and approved by Istanbul University-Cerrahpaşa Faculty of Medicine. Written informed consent for participation was not required for this study in accordance with the national legislation and the institutional requirements.

## Author contributions

UY, SK, and ST collected data. UY performed the statistical analysis. UY and AE wrote the manuscript. AE edited the manuscript. All authors contributed to the article and approved the submitted version.

## Conflict of interest

AE has received advisory board and speaker bureau honoraria from Novartis, Bristol-Myers Squibb, and Pfizer.

The remaining authors declare that the research was conducted in the absence of any commercial or financial relationships that could be construed as a potential conflict of interest.

## Publisher’s note

All claims expressed in this article are solely those of the authors and do not necessarily represent those of their affiliated organizations, or those of the publisher, the editors and the reviewers. Any product that may be evaluated in this article, or claim that may be made by its manufacturer, is not guaranteed or endorsed by the publisher.

## References

[1] Hochhaus A Baccarani M RT S Schiffer C JF A Cervantes F. European LeukemiaNet 2020 recommendations for treating chronic myeloid leukemia. Leukemia (2020) 34(4):966–84. doi: 10.1038/s41375-020-0776-2 PMC7214240 32127639

[2] Duncan N Deekes N FitzGerald D Ng TWT Raghavan M. Models of care for chronic myeloid leukemia patients during the COVID-19 pandemic in the united kingdom: Changes in patient attitudes to remote consultations and future implications. EJHaem (2021) 2(3):394–9. doi: 10.1002/jha2.220 PMC8242739 34226902

[3] Abruzzese E Luciano L D'Agostino F MM T Pane F De Fabritiis P. SARS-CoV-2 (COVID-19) and chronic myeloid leukemia (CML): A case report and review of ABL kinase involvement in viral infection. Mediterr J Hematol Infect Dis (2020) 12(1):e2020031. doi: 10.4084/mjhid.2020.031 32395220 PMC7202347

[4] Breccia M Abruzzese E Accurso V Attolico I Barulli S Bergamaschi M. COVID-19 infection in chronic myeloid leukaemia after one year of the pandemic in Italy. A Campus CML Rep Br J Haematol (2022) 196(3):559–65. doi: 10.1111/bjh.17890 PMC8652631 34636033

[5] Yılmaz U Pekmezci A Gül Y Eşkazan AE. COVID-19 in chronic-phase chronic myeloid leukemia patients: A single-center survey from Turkey. Turkish J Hematol (2021) 38(1):79. doi: 10.4274/tjh.galenos.2020.2020.0472 PMC7927441 32964857

[6] Rea D MJ M JE C Jiang Q KB P Ongondi M. COVID-19 in patients (pts) with chronic myeloid leukemia (CML): results from the international CML foundation (iCMLf) CML and COVID-19 (CANDID) study. Blood (2020) 136:46–7. doi: 10.1182/2Fblood-2020-140161

[7] Pagnano KB EH P JR N LD DS Delgado N Moiraghi B. COVID-19 in chronic myeloid leukemia patients in Latin America. Leuk Lymph (2021) 62(13):3212–8. doi: 10.1080/10428194.2021.1950709 34254886

[8] Claudiani S JF A EL P Marchesin F Katsanovskaja K Palanicawandar R. Durable humoral responses after the second anti-SARS-CoV-2 vaccine dose in chronic myeloid leukaemia patients on tyrosine kinase inhibitors. Br J haematol (2021) 197(1):e1–4. doi: 10.1111/bjh.18001 34923623

[9] Harrington P KJ D Radia D O’Reilly A HP L Seow J. Single dose of BNT162b2 mRNA vaccine against severe acute respiratory syndrome coronavirus-2 (SARS-CoV-2) induces neutralising antibody and polyfunctional T-cell responses in patients with chronic myeloid leukaemia. Br J Haematol (2021) 194(6):999–1006. doi: 10.1111/bjh.17568 34085278 PMC8239833

[10] Delgado N Torres A. What do we currently know about chronic myeloid leukemia (CML) and COVID-19? Curr Oncol Rep (2022) 26:1–6. doi: 10.1007/s11912-021-01169-w PMC8881701 35218499

[11] Claudiani S. Is COVID-19 less severe in CML patients than in those with other haematological cancers? Br J haematol (2021) 196(3):471–2. doi: 10.1111/bjh.17927 PMC8653325 34708401

[12] Galimberti S Petrini M Baratè C Ricci F Balducci S Grassi S. Tyrosine kinase inhibitors play an antiviral action in patients affected by chronic myeloid leukemia: A possible model supporting their use in the fight against SARS-CoV-2. Front Oncol (2020) 10:1428. doi: 10.3389/fonc.2020.01428 33014780 PMC7493657

[13] Yilmaz U Eskazan AE. Moving on from 2013 to 2020 European LeukemiaNet recommendations for treating chronic myeloid leukemia: what has changed over the 7 years? Expert Rev Hematol (2020) 13(10):1035–8. doi: 10.1080/17474086.2020.1813564 32814447

